# Rapid Health Needs Assessment Experience in 11 August 2012 East Azerbaijan Earthquakes: A Qualitative Study

**DOI:** 10.1371/currents.dis.308f6140d54f78fd1680e2b9e6460ae3

**Published:** 2014-07-07

**Authors:** Javad Babaie, Shandiz Moslehi, Ali Ardalan

**Affiliations:** Department of Disaster Public Health, School of public Health, Tehran University of Medical Sciences, Tehran, Iran; Department of Disaster and Emergency Health, National Institute of Health Research, Tehran University of Medical Sciences, Tehran, Iran; Department of Disaster Public Health, School of public Health, Tehran University of Medical Sciences, Tehran, Iran; Department of Disaster and Emergency Health, National Institute of Health Research, Tehran University of Medical Sciences, Tehran, Iran; Department of Disaster & Emergency Health, Iran's National Institute of Health Research; Department of Disaster Public Health, School of Public Health, Tehran University of Medical Sciences, Tehran, Iran; Harvard Humanitarian Initiative, Harvard University, Cambridge, Massachusetts, USA

**Keywords:** disaster, Earthquake, East Azerbaijan, Rapid Health Needs Assessment

## Abstract

Introduction: In disasters, health care providers need to find out the essential needs of the affected populations through Rapid Health Needs Assessment (RHNA). In East Azerbaijan earthquakes, a rapid assessment was performed by the provincial health system. The main purpose of this study was to explore the RHNA challenges. Methods: In this qualitative study (Grounded theory), data was collected through semi-structured interviews with purposely selected health care workers. The data collection process continued until data saturation. All interviews were recorded and then transcribed. The Colaizzi's descriptive method was used to analyze the data. Results: The themes emerged from the analysis of the interviews were: 1) Logistic problems 2) Lack of RHNA tools 3) Inherent difficulty of RHNA in disaster situations 4) Lack of preparedness and 5) Lack of coordination between different organizations. These challenges result in inapplicable use of RHNA results. Conclusion: The most important challenge in this RHNA process was the lack of East Azerbaijan health center preparedness. Although they were familiar with the importance of RHNA, they did not have any plans for conducting RHNA.

## Introduction

Natural hazards are common phenomena all around the world. They threaten the lives of millions of people 1 . In the first days following a disaster, health providers need valid and reliable information for decision making, resource allocation, and providing the most essential and effective services. To work efficiently in such situations 2 , the first important task is to conduct a rapid health needs assessment (RHNA) 3 ^,^ 4. There are many examples that show health care providers have performed RHNA in the first days following disasters 5 ^-^ 8 to assess the health needs of the affected community, to prevent or reduce the mortality rate, to plan for health care equity 9, to establish priorities, and to determine where the resources should be allocated 10.

The twin earthquakes in Iran that shook East Azerbaijan, northwest of Iran, on 11 August 2012 (Ahar, Herris and Varzeghan districts) 11 destroyed 272 villages, affected more than 85466 people, claimed the lives of about 300, and injured 3309 people. In the first hours after the earthquake, Tabriz University of Medical Sciences deployed four teams for RHNA. The RHNA was completed 5 days after the earthquake, however there were some challenges in performing RHNA.

Because it was the first experience of East Azerbaijan health system the main purpose of this study is to provide an overview of the needs and health status of the most affected community in Azerbaijan and to assess the challenges with RHNA.

## Methods

***Design***

In this study, our paradigm was to perform a qualitative study because we did not have any theories for the main study question. We used the grounded theory as the methodology to discover the experiences of the health workers who were involved in conducting RHNA in East Azerbaijan due to lack of knowledge in this area and also because of the social process in disaster prone areas.

Therefore, we found the local level grounded theory was the best choice to answer our study question. Because these areas were limited we named it “local level grounded theory approach”. It is a substantive product of grounded theory and it depends/relates to a limit area.

One of the main aims of the grounded theory is to generate hypotheses, theories, and tentative models based on empirical data. This approach is considered to be suitable for exploring a known area from a fresh perspective 18 ^,^ 19 ^,^ 20 .

***Study setting***

The study was conducted in East Azerbaijan Province, northwest of Iran. This region experienced two earthquakes with the magnitudes of 6.4 and 6.3 on the Richter scale on 11 August 2012. East Azerbaijan is divided into 20 districts. The earthquakes affected three districts of north of East Azerbaijan, Heris, Ahar and Varzeghan. These three districts have 557 rural areas with 135656 inhabitants of which 250 villages with 82148 inhabitants were affected by the earthquakes.

***Study participants and data collection***

The study participants consisted of health workers who were actively involved in the RHNA process. The inclusion criterion for selecting interviewees was that the health workers had to have at least one experience in RHNA in the first five days after the earthquake. These participants were purposely selected from East Azerbaijan health centers and the health centers of the three affected districts. We excluded the participants who did not have a firsthand and practical experience in the RHNA process.

Data was collected using semi-structured deep interviews and analyzed based on a grounded theory approach. The interviews took 45-60 minutes for each person. The participants were asked to tell their experiences and the challenges of conducting RHNA. All interviews were conducted in their native language (Turkish language that is spoken in East Azerbaijan). The interviews were done in the participants’ offices. Verbal consent was obtained, and all participants were informed that they could refuse to participate or withdraw the interviews at any time. The data collection process continued to clarify and develop the codes, categories, concepts and to saturate them.

***Data analysis***

All interviews were recorded, transcribed and translated by investigators. The Colaizzi’s descriptive method was used to analyze the data. The authors only expressed the views of participants and used member check, peer check, and expert check for trust- worthiness of the study (investigator triangulation).

## Results

About 1 hour after earthquakes, 3 RHNA teams were deployed to the areas with a pen and paper. The teams were composed of environmental health officers of Tabriz health center. Their task was to collect preliminary data. These teams could only determine where the earthquakes occurred and which areas were affected. On the next day, environmental health officers from the health centers all over the country were deployed to the affected areas as RHNA operational teams. The formal RHNA process started on the first day after the earthquakes. They used the RHNA checklist that was provided in "Emergency Operation Plan: A National Guideline"^12^ for collecting data. Because it was too long and included many details, they concluded that it had to be revised. We note that the validity of this revised tool was not checked because of the limited time. The revised version of the RHNA checklist was then used.

The completed RHNA checklists were collected and delivered to the provincial health center managers. Because many villages were affected, it was not possible for RHNA teams to go to all villages and perform the assessment themselves. Thus, from the second day forward, the checklists were completed by any health worker who was deployed to the earthquake areas eg. nurses, physicians. Finally, after about a week, preliminary results were obtained (although incompletely). Varzeghan RHNA forms were not completed. Table 1 highlights the preliminary results of RHNA.

**Table 1: Preliminary results of RHNA of East Azerbaijan districts:** *In Iran's health system, primary health care is provided in different levels. The first level is "Health houses" which are located in villages and are staffed by trained community health workers "Behvarz". "Rural health centers" include at minimum a general physician and some health technicians. They are responsible for providing medical services to patients, and to support and supervise health houses.

	Varzeghan		Herris		Ahar	
No. of Houses	Destroyed	Safe	Destroyed	Safe	Destroyed	Safe
	2367	880	3104	455	312	139
Area Population	Healthy	Injured	Healthy	Injured	Healthy	Injured
	2337	9567	7802	6384	2414	1685
No. of Death	?		106		35	
No. of Injured People	?		130		39	
No. of Active Rural Health Center	?		2		5	
No. of Active Health House^*^	?		6		6	
Percentage of Household Accessibility to the Safe Water	?		28%		49%	

Thirteen workers from East Azerbaijan and the health centers of the three affected districts participated in semi- structured interviews. These interviewed participants were actively involved in RHNA. They were 3 females and 10 males. All of them had university-level education.

Six themes emerged from the analysis of the interviews including: 1) Logistic problems for caring out of RHNA; 2) Lack of appropriate data gathering tool; 3) Inherent difficulty of RHNA in disaster situations; 4) Lack of preparedness; 5) Executive challenges and lack of coordination between different organizations. These challenges meant the results of the RHNA process were not applicable and useful.

Managers and workers of East Azerbaijan health centers were familiar with the role of RHNA in understanding the current situation analysis, estimation of public health needs, and planning for providing these needs. Thus, the RHNA process started as the first response measure. One of the participants said:

" … It (RHNA) was very important. We want to establish our health facilities to provide primary health care but we don’t know how many people are affected, what they need, and how many health relief/response teams we need. Information is vital for planning. Thus, we tried to do RHNA as soon as possible and we did it…" 

According to the answers, all of those who were involved in the RHNA process understood the necessity of RHNA. The investigators of this project do not have any doubts about it.

**1. Logistic problems for carrying out of RHNA**

Logistic problems were a big challenge because the devastated areas were very vast and more than 250 villages were destroyed in three big districts.

A participant said: *"…Three districts were affected. Most of our personnel, even in Tabriz, were affected. They had some difficulties with their families. We could deploy only three teams on the first days. We did not have enough human resources to assemble more teams. They were our environmental health personnel. We told them to go wherever they could and collect whatever they could…"*

**2. Lack of appropriate data gathering tool**

The national "Emergency Operation Plan: EOP (12)", was developed only 6 months earlier than these earthquakes and it was sent to all provinces, but none of public health centers staff had studied it. It included a rapid needs assessment check list. However, in the mentioned EOP, there was no guideline on how to do RHNA. In the first stage, this checklist was printed and distributed in teams. The teams started to complete it immediately. At first, they thought this form was appropriate but very soon, they learned that it was very detailed, its completion was very time consuming and they already had some of the data. Then, they immediately revised it. Thus, a different version of RHNA checklists was used. Consequently, their results became different. Lack of an appropriate tool was a big problem.

A study participant said: *"These checklists aren't real. They should be revised. We couldn't collect some data that were included in them".* Another participant said: *"There was no necessity to use the resources to collect things that we already had. In addition, different departments of the provincial health centers had their own special information. For example, the communicable diseases department knows how many Tb [Tuberculosis]**** patients there are and where they are, or they have the endemic diseases of every village in detail."*

A participant said: "*However, understanding the situation and emerged needs are the first steps in every process planning, but we had most of the information in checklists. The only thing that we needed was the number of affected villages and people ".*

**3. Inherent difficulty of RHNA in disaster situations**

The participants of the study believed that the RHNA process was challenging. In disasters, infrastructures of the community are damaged and access to the affected areas is hindered. Informant resources are affected and much of the existing data is destroyed. Even if the residents of the affected areas are not affected, they are in a situation that cannot respond to our requests.

*"Our health center was damaged. We couldn't enter it. There were still aftershocks ".* Another said: *"The process of data collection, summarizing and analyzing is very time consuming. It needs a special and trained expert and some tools."* The other believed*:" Over the time, the type of needs changed. The results of* *RHNA that were done on the first day were completely different from the results of the fifth day. In addition, all of the data was estimations. They weren’t accurate."*

**4. Lack of preparedness**

All participants believed that they were not adequately prepared. Some of them had some RHNA related experiences in Bam earthquake and the Iran-Iraq war but none of them were actively involved in RHNA.

One of the interviewees said: *"There was a big problem. Those who wanted to do RHNA were not trained for even an hour"*. Another participant said: *"Because different people completed the RHNA forms, they were completed differently. Forms were sent to workers and they were required to complete them without any prior training." *And another participant stated: *"We didn't have any plans for RHNA, and there was no one responsible for completing it."*

**5. Lack of coordination between different organizations**

There were some executive problems in the RHNA process. Approximately all of the governmental organizations started their own RHNA. Although most of the required data was similar, there was no coordination between the organizations to share them.

One of the participants said: *"Many organizations that were involved in the response phase had started their RHNA. Although most of the data that had to be collected was similar, there was no coordination between them. Also, because they had their own special literature, they didn't understand each other."*

According to the participants, although great efforts were made to collect information and a lot of resources were spent, the results of RHNA were not used for planning because of the above-mentioned problems.

A participant said: *"When the results of RHNA were prepared, there was no need for them. Because our public health relief/response teams were established and they started providing services." *A manager in a provincial health center said: *"Until now (1 month after the earthquake), the results of RHNA are not prepared. I am trying to get them. Half of them are here but I don’t know where the other half is" "We couldn’t rely on data. It seemed that they were not completed accurately."*

Figure 1 highlights the RHNA process in response to East Azerbaijan earthquakes.

**Figure:**
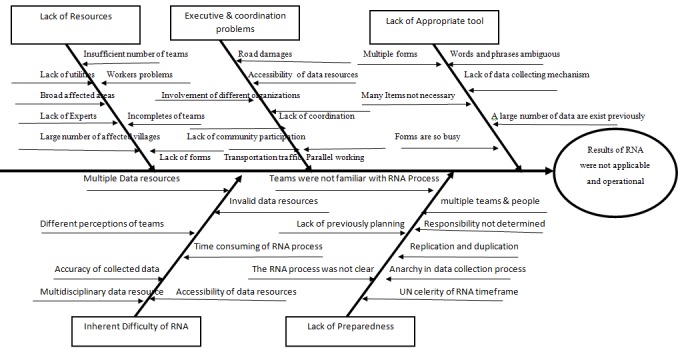

## Discussion

Immediately after the disaster, relief/response provider organizations need to analyze the current situation and set priorities. Thus, rapid needs assessment following the disaster is one of the first activities 13 which should be performed as soon as possible. In East Azerbaijan earthquakes, this process was noticed, done immediately after the earthquake and carried out in 5 days. The RHNA processes were completed in Ahar and Herris, but it was never completed for Varzegan. The main objective of this study was to investigate the challenges of the RHNA process in East Azerbaijan two earthquakes.

Analysis of the interviews showed that the PHC managers and workers knew the importance of RHNA but they had some problems with performing it. The main challenges were lack of resources, lack of appropriate RHNA tools, inherent difficulty of data collection in chaotic environments, lack of preparedness, and coordination challenges.

According to the results of RHNA, due to East Azerbaijan twin earthquakes, more than 5783 houses were destroyed, 169 persons were injured, and 141 persons died. Seven health centers were active, drinking water accessibility was 28% in Herris and 49% in Ahar, and 12 health houses were active as well. Although later more accurate results showed that they were not correct. However, thsi inaccuracy of RHNA results was not unexpected.

Unpreparedness of East Azerbaijan health center workers was the most important challenge of this RHNA process. Although they knew the importance of RHNA, they did not have any plans for carrying out RHNA. Thus, they did not have any expert and trained teams. Even the personnel who wanted to perform RHNA did not have any formal or informal education before deployment. It is clear that the results of the efforts of such teams will not be accurate. Ciotton et al reported this challenge. They mentioned that in many disasters, the RHNA process is performed by unprepared and untrained personnel although the existing references emphasize that it should be done by trained teams 14. Using different participants for data collection was not a good experience because they were not taught how to collect the data. Some of them had no firsthand experience in data collection, some of them did not have any knowledge about the field and some were not familiar with RHNA. The results of this study highlight the importance of preparedness for conducting RHNA.

Although Iran is a disaster prone country and has been affected by catastrophic disasters several times 15, there is no accepted tool for collecting basic data in disasters and emergencies. However, there are some RHNA tools/questionnaires 12 ^,^ 14 but references emphasize that such tools should be developed by communities with regards to their characteristics. A checklist/questionnaire was recently prepared in Iran but according to the participants of this study, it was not appropriate and had to be revised urgently. One of the most important challenges that participants experienced was the lack of such a tool. Although they tried to develop a new version, they believed that it was not perfect either. Thus, the collected data was not perfect and could not help the responders.

A systematic review in 2010 showed that most of the RHNAs in the studied disasters started 3-14 days after the disaster 14 ^,^ 5. However, the RHNA in East Azerbaijan started immediately; thus, it was the main strength point.

There are many methodologies and sampling procedures (such as cluster sampling) which can facilitate the RHNA process 15. Such methodologies were used in the RHNA process in Colorado & San Antonio after Katrina 5 ^,^ 6 but none of these tools and sampling methods was used in this RHNA. The RHNA teams assessed all of the affected villages, which can lead to wasting the resources.

No one was responsible for managing the RHNA process. This challenge led to anarchy in data collection, summarization, and application in planning. Lack of coordination in conducting RHNA is an important problem in other countries, too 16.

Some studies have shown that logistics and security are important obstacles in performing RHNA. These are 2 main effective factors for working efficiently in disasters 17. Fortunately, in Ahar, Herris and Varzeghan, there were no problems with security although lack of resource was a big challenge.

## Conclusion

RHNA is the most important component of every disaster response plan. It determines the needs and sets priorities. Therefore, responsible organizations should be equipped, trained, and prepared for performing it. Iran is vulnerable to many disasters but unfortunately it does not have any accepted tools for RHNA. Almost none of the medical universities have an expert team. It is necessary to develop appropriate tools as soon as possible and to train experts to respond in the field.

## Key words

Disaster, Earthquake, Rapid assessment, Iran

## Correspondence

Ali Ardalan. Email: aardalan@tums.ac.ir, ardalan@hsph.harvard.edu
